# Chemotherapy for elderly colorectal cancer patients at a tertiary hospital in South Africa

**DOI:** 10.11604/pamj.2020.37.100.18515

**Published:** 2020-09-29

**Authors:** George Pupwe, Owen Ngalamika, John Akudugu

**Affiliations:** 1 Division of Clinical and Radiation Oncology, Tygerberg Hospital, Cape Town, South Africa,; 2 Cancer Diseases Hospital, Lusaka, Zambia,; 3 Dermatology and Venereology Section, University Teaching Hospital, University of Zambia School of Medicine, Lusaka, Zambia,; 4 Division of Radiobiology, Stellenbosch University, Cape Town, South Africa

**Keywords:** Elderly population, colorectal cancer, chemotherapy, toxicity, progression free survival, overall survival

## Abstract

**Introduction:**

surgical treatment of colorectal cancer (CRC) in elderly patients has improved, but data on the tolerability and benefits of adjuvant and palliative chemotherapy in this growing population remains scarce.

**Methods:**

we conducted a retrospective study to compare chemotherapy-associated toxicities in CRC patients aged < 70 years and ≥ 70 years at Tygerberg Hospital (South Africa). We also assessed tumor-related mortality, progression free survival (PFS), and overall survival (OS) including predictive factors of OS.

**Results:**

a total of 50 patients received either adjuvant or palliative chemotherapy. There was no difference in overall toxicity between the two groups. Out of the 50 patients, 8 (16%) had Grade 3-4 toxicity. 4 of these patients made up 15% of the < 70 years age group, whereas the other 4 made up 17% of the ≥ 70 years age group. The mean follow-up time was 47.5 months (95% CI 41.5 - 53.5 months). The 5-year over-all survival rate for stage II and III patients < 70 years and ≥ 70 years were 80.9% and 69.5%, respectively, and not significantly different (P = 0.52). Furthermore, the 5-year progression-free survival rates of the < 70 and ≥ 70 age groups were 70.7% and 58.8%, respectively, and also not statistically significantly different (P = 0.49). For stage IV patients, there were no significant differences in survival between the two age groups.

**Conclusion:**

the benefits from adjuvant and palliative chemotherapy for elderly CRC patients are similar to that of younger patients. Therefore, standardized adjuvant and palliative chemotherapy is recommended for elderly patients.

## Introduction

Colorectal cancer (CRC) accounts for 10% of cancer diagnoses and deaths. It is the third most common cause of cancer deaths in the world [1]. CRC usually affects older people, with 67 to 75% being ≥ 65 years [2]. The average age at diagnosis is 72 years, with 70% of patients > 65 years and 40% older than 75 years [3, 4]. The result is a high proportion of elderly CRC patients that require treatment [5].

Treatment of CRC is based on staging. The standard of treatment for stage I and II colon cancer is surgery, although a specific subset of stage II colon cancer also require adjuvant chemotherapy [6]. The standard of care for stage III colon cancer is surgery, followed by adjuvant chemotherapy [7, 8]. Stage IV CRC is best treated with systemic chemotherapy to prolong survival and also to help improve symptoms and quality of life. A systematic review undertaken by the Colorectal Cancer Collaborative Group compared palliative chemotherapy with supportive care and it found an improvement of 3.7 months in median survival in favor of the chemotherapy arm [9].

The majority of CRC patients referred to the Department of Clinical and Radiation Oncology, Tygerberg Hospital (South Africa), with histologically confirmed adenocarcinoma of the colon/rectum present with either locally advanced or metastatic disease. Due to the advanced stage at presentation, most of the newly diagnosed CRC patients undergo systemic chemotherapy. However, there are no published data on the tolerability and benefits of adjuvant and palliative chemotherapy at our cancer center. This study aimed at determining toxicity and outcomes of elderly CRC patients that undergo chemotherapy at Tygerberg hospital in South Africa.

## Methods

**Study design and setting:** a retrospective study conducted at the Department of Clinical and Radiation Oncology at Tygerberg Hospital (South Africa).

**Study population and Inclusion/Exclusion criteria:** all patients referred with histologically proven adenocarcinoma of the rectum/colon who received chemotherapy between January 2009 and December 2013 were included in the study. Two hundred and sixty files were reviewed. 210 files of patients who did not receive chemotherapy or those who received chemotherapy but had incomplete records (data/files missing) were excluded. The remaining 50 files were evaluated in this study.

**Data collection:** the relevant data were collected from the therapy folders. Data from imaging studies were collected from the Picture Archiving and Communications System (PACS) system, and the laboratory results were collected from the DISALAB program. Data were collected using the patient´s folder number only and no name or identifiable information was used. The collected data included the age, gender, tumor characteristics (localization, grade, extension, stage), treatment, comorbidities, HIV status, performance status (PS), weight loss, alkaline phosphatase, the number of metastatic sites, time since diagnosis to start of treatment, toxicities due to chemotherapy, and if the chemotherapy course was completed or not. The reasons why the chemotherapy was stopped, if applicable, and also the cause of death were also recorded. The toxicities associated with chemotherapy were recorded according to Common Toxicity Criteria.

**Statistical analysis:** all the data were collected in coded questionnaires and analyzed using the GraphPad Prism software (San Diego, USA). Progression free and overall survival rates, as well as predictive factors for overall survival were compared using Kaplan-Meier curves.

**Ethical considerations:** approval was granted for the study by Human Research Ethics Committee of University of Stellenbosch (Ethics Ref: S15/02/040). Patient records were reviewed with utmost confidentiality. The individual´s data set was allocated a unique study number and the data were stored in a secure office, as well as on a computer to which only the principal investigator had access via a password.

## Results

### Patients and tumor characteristics

50 patients were recruited into the study, 29 (58%) were male and 21 (42%) were female. Age at diagnosis ranged from 28 to 78 years (mean of 62 years). Twenty-seven (54%) of the patients were aged < 70 years, and 23 (46%) were aged ≥ 70 years. Most of the patients, 47 (94%), had World Health Organization (WHO) performance status (PS) of 1. Three (6%) patients had WHO PS of 2. Data for tumor characteristics and treatment options are summarized in Table 1. Of the 50 patients, 22 had colon cancers and subsites included the left, right and sigmoid colon. The remainder of the patients had rectal cancer. Most patients (36 out of 50) had Grade 2 adenocarcinoma. Eight patients had grade 1, and 2 patients had grade 3 tumors. Four patients had tumors of unknown grade. Out of the 50 patients, 28 patients had stage III disease at diagnosis. Ten patients had stage II disease and 12 patients had stage IV disease. All 50 patients received different regimens of chemotherapy which included 5-FU/LV bolus, 5-FU infusion and Capecitabine. Two patients did not complete the 6 cycles of chemotherapy due to neutropenic sepsis. Forty-six patients had surgery and 34 had radiotherapy. As for comorbidities, 21 patients had hypertension, 3 had pulmonary tuberculosis, 2 had ischemic heart disease, 3 had deep vein thrombosis, 3 had diabetes mellitus type 2, whereas 18 had no comorbidities.

**Table 1:** patient characteristics and therapeutic approach

Age group	< 70 yrs (n = 27)	≥ 70 yrs (n = 23)
**Males**	14(52%)	15(65%)
**Site**		
Colon	13(48%)	9(39%)
Rectum	14(52%)	14(61%)
**Differentiation**		
I	5(19%)	3(13%)
II	18(67%)	18(78%)
III	1(4%)	1(4%)
Unknown	3(11%)	1(4%)
**Tumor stage at diagnosis**		
II	6(22%)	4(17%)
III	15(56%)	13(57%)
IV	6(22%)	6(26%)
**Treatment**		
Surgery	24(89%)	22(96%)
Radiotherapy	17(63%)	17(74%)
Chemotherapy	27(100%)	23(100%)
**Weight loss**		
<5%	22(81%)	17(74%)
> 5%	5(19%)	6(26%)
**Comorbidities**		
None	15(56%)	3(13%)
Hypertension	7(25%)	14(61%)
Pulmonary tuberculosis	1(4%)	2(9%)
Heart disease	1(4%)	1(4%)
Diabetes mellitus	0	3(13%)
Deep vein thrombosis	3(11%)	0

### Tumor related mortality

Tumor related mortality was assessed using univariate and multivariate analysis. Univariate analysis demonstrated that patients with rectal cancer, stage III and stage IV disease, and those who had surgery had a higher risk of mortality, as shown in Table 2. Gender, age, comorbidity, and radiotherapy did not play a significant role in tumorrelated mortality. From multivariate analysis, only patients with stage III and stage IV disease were at a higher mortality related risk compared to stage II patients, with p-values being 0.17 and 0.009 respectively. There was no difference in tumor related mortality risk in relation to age, gender, surgery, radiotherapy, and subsite of disease.

**Table 2:** univariate analysis on risk factors for tumor related mortality

Characteristic	Hazard ratio^*^	P-value	95% CI
Male	1.0		
Female	1.08	0.86	0.45-2.57
Colon	1.0		
Rectum	0.38	0.03	0.16-0.92
Stage II	1.0		
Stage III	6.14	0.007	1.62-23.20
Stage IV	13.32	<0.0001	3.62-49.17
< 70 years	1.0		
≥ 70 years	0.75	0.67	0.13-2.79
No comorbidity	1.0		
Comorbidities	1.48	0.92	0.57-3.81
No surgery	1.0		
Surgery	0.1	<0.0001	0.03-0.32
No radiotherapy	1.0		
Radiotherapy	0.94	0.88	0.38-2.32

* Hazard ratio of 1.0 implies reference value.

### Toxicities from chemotherapy

8 (16%) of all the recruited patients had Grade 3-4 toxicity from chemotherapy. 4 of these patients were < 70 years and 4 were ≥ 70 years, representing 15% and 17% of patients in these groups respectively. 36 (72%) of all the recruited patients had Grade 1-2 toxicity, of which 17 were < 70 years and 19 were ≥ 70 years, accounting for 63% and 83% in these two age groups respectively. Neutropenic sepsis was observed in 6 patients. 3 of these patients were < 70 years while 3 patients were ≥ 70 years, representing 11% and 13% of these age groups respectively. Table 3 shows the distribution and types of toxicities experienced for this population by age group. Both age groups experienced comparable toxicity.

**Table 3:** distribution and types of toxicity by age

Toxicity type	<70 yrs. (n=27)	≥ 70 yrs. (n = 23)
**Nausea and vomiting**		
Grade 0	17(63%)	14(52%)
Grade 1-2	9(33%)	8(30%)
Grade 3-4	1(4%)	1(4%)
**Diarrhea**		
Grade 0	18(67%)	13(57%)
Grade 1-2	8(30%)	9(39%)
Grade 3-4	1(4%)	1(4%)
**Dermatitis**		
Grade 0	27(100%)	21(91%)
Grade 1-2	0	2(8%)
Grade 3-4	0	0
**Hand + foot syndrome**		
Grade 0	25(93%)	21(91%)
Grade 1-2	0	0
Grade 3-4	2(7%)	2(8%)
**Neutropenic sepsis**		
None	24(89%)	20(87%)
Present	3(11%)	3(13%)

### Survival

**Figure 1:**
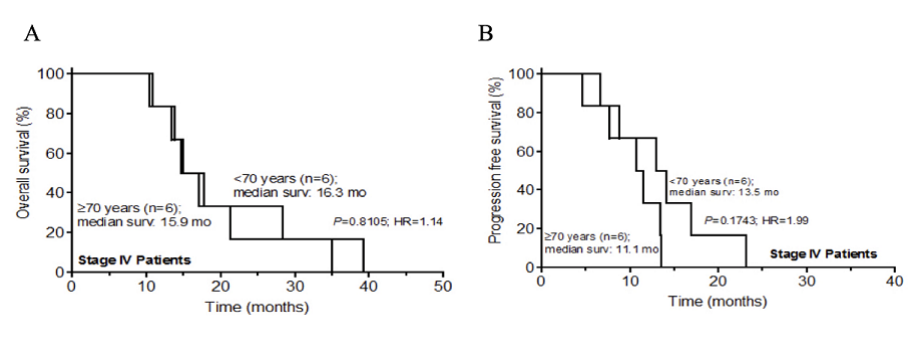
A) overall survival rates for stage IV colorectal cancer patients in age groups <70 years and ≥ 70 years; B) progression free survival rate for stage IV colorectal cancer patients in age groups < 70 years and ≥ 70 years

**Figure 2:**
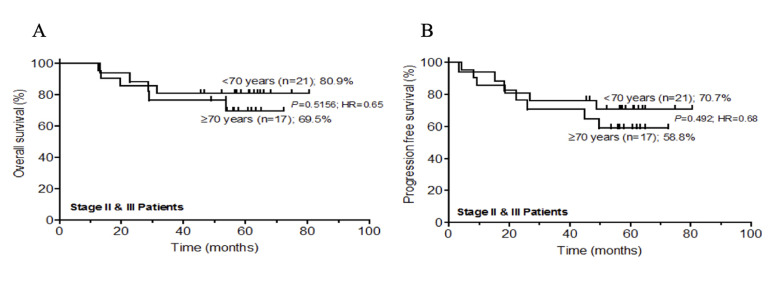
A) overall survival rate at 5 years for stage II and III colorectal cancer patients in age groups < 70 years and ≥ 70 years; B) progression free survival rate at 5 years for stage II and III colorectal cancer patients in age groups < 70 years and ≥ 70 years

The progression free survival (PFS) and overall survival (OS) were measured using Kaplan-Meier method. The mean follow-up time was 47.5 months (range: 14.4-80.8 months). There was no significant difference in overall survival between both groups for stage IV patients (Figure 1 A). There were no survivors beyond 40 months of follow up. The median survival was 16.3 months (for < 70 years) and 15.9 months (for = 70 years); P=0.8105; HR=1.14(95% CI: 0.35-3.81). Figure 1 B shows PFS for stage IV patients, showing no significant difference in survival between the two groups. There were no progression free survivors beyond 23 months of follow up. The median PFS was 11.1 months (for < 70 years) and 13.5 months (for = 70 years); P=0.1743; HR=1.99(95% CI: 0.66-9.67). Figure 2 A shows OS for stage II and III patients. There was no significant difference in survival between the two groups. The 5-year overall survival rate for patients < 70 years and ≥ 70 years were 80.9% and 69.5%, respectively; P = 0.5156; HR = 0.65(95% CI: 0.17-2.41). Figure 2 B shows PFS for stage II and III patients, and shows no significant difference in survival between the groups. The 5-year progression free survival rate for patients < 70 years and ≥ 70 years were 70.7% and 58.8%; P = 0.4920; HR = 0.68(95% CI: 0.23-2.04). Sixty-eight and 84% of stage II and III patients in the < 70 and ≥ 70 years age groups presented with less than 5% weight loss, respectively. For stage IV patients, the corresponding proportions for the two age groups were 75% and 100%. In addition, 84% and 100% of stage II and III patients in the < 70 and ≥ 70 years groups, respectively, had a performance status (PS) of 1. All stage IV patients had a PS of 1.

## Discussion

This study compares toxicities and outcomes of CRC chemotherapy between patients less than 70 years of age and those 70 years or older. Most of the patients had either locally advanced disease or metastatic disease, and were eligible for chemotherapy. The reason for late presentation may be due to late diagnosis or a poor referral system in a resource limited environment. 32% of our study population was >75 years of age. Kohne *et al*. reported the average age of patients diagnosed with CRC in the United States in 2008 to be 71 years [3]. In recent years, elderly patients are making up a significant proportion of CRC studies compared to the past due to an increase in the number of patients undergoing curative resections for CRC, as well as an associated decrease in post-operative mortality [3]. However, a few studies have still shown underrepresentation of elderly patients above 75 years of age [10, 11]. In our study, there was no statistically significant difference in tumor-related mortality between patients below 70 years of age and patients that were 70 years and above. This is contrary to Serra-Rexach *et al*. who found that younger patients had a longer tumor-specific survival time than older patients (36.41 months versus 26.05 months) [11]. In our study, we also found that patients with stage III and IV disease were at a higher risk of tumor-related mortality, and that surgery did not affect the tumor-related mortality risk. This is similar to the study by Serra-Rexach *et al*. who reported that patients with stage III and IV disease and did not undergo surgery had a high tumor-related mortality risk [11]. In addition, we also found that there was no significant difference in the 5-year overall survival and progression-free survival for stage II and III patients < 70 years and ≥ 70 years of age.

We also analyzed the incidence of side effects such as nausea and vomiting, hand and foot syndrome, dermatitis, diarrhea, and neutropenic sepsis. We found that both age groups experienced similar toxicity. This is consistent with the study by Sargent *et al*. who reported that elderly patients >70 years did not experience more side effects than younger patients except for leukopenia [12]. We, however, did not compare the severity of these side effects between different types of chemotherapy regimens as other studies have done in the past. We mainly use 5-FU continuous infusion or 5-FU/LV bolus therapy due to the limited resources available.

Our data suggest that chemotherapy in elderly patients with CRC is well tolerated as nearly half of our study population was older than 70 years. This is consistent with the published literature [10, 11]. Careful selection of patients is probably more important in the elderly population [11]. In this study, 94% of patients had an Eastern Cooperative Oncology Group Performance Status (ECOG PS) of 1. In the palliative setting, patients with a good PS are the ones likely to benefit from chemotherapy, and treatment should start as soon as possible before their PS deteriorates [13].

The absence of significant differences in overall survival between the two age groups, regardless of disease stage, may be attributed to the finding that the degree of weight loss and PS for both groups was comparable and that the study cohort was reasonably healthy. Put together, our study shows that weight loss and performance status are the two major factors that appear to be independently associated with a better OS.

## Conclusion

Stable elderly colorectal cancer patients benefit, at least to the same extent, from adjuvant and palliative chemotherapy as younger patients in our setting. Therefore, we advocate the use of standardized adjuvant and palliative chemotherapy for elderly patients. Age should not influence the decision to offer adjuvant or palliative chemotherapy to older patients. Assessment of PS and weight loss are a useful guide in decision making for difficult cases.

### What is known about this topic

- Colorectal cancer is the third most common cause of cancer deaths in the world;
- The standard of treatment for stage I and II colon cancer is surgery, although a specific subset of stage II colon cancer also require adjuvant chemotherapy; the standard of care for stage III colon cancer is surgery, followed by adjuvant chemotherapy; stage IV CRC is best treated with systemic chemotherapy to prolong survival and also to help improve symptoms and quality of life;
- The average age at diagnosis of colorectal cancer is 72 years. The elderly population is largely under-represented in research studies.

### What this study adds

- Stable, elderly, colorectal cancer patients benefit at least to the same extent from adjuvant and palliative chemotherapy as younger patients in our setting.
- Age should not influence the decision to offer adjuvant or palliative chemotherapy to older patients;
- Assessment of performance status and weight loss are a useful guide in decision making for difficult cases.
